# Absolute and Relative Morbidity Burdens Attributable to Various Illnesses and Injuries Among Non-Service Member Beneficiaries of the Military Health System, 2023

**Published:** 2024-07-20

**Authors:** Armed Forces Health Surveillance Division Defense Health Agency, US

## Abstract

**What are the new findings?:**

Mental health disorders accounted for the largest proportions of the morbidity and health care burdens that affected the pediatric and younger adult beneficiary age groups of nonservice member beneficiaries of the Military Health System in 2023. Among adults aged 45-64 years and adults aged 65 years and older, musculoskeletal diseases accounted for the most morbidity and health care burdens. With almost all health care for Medicare-eligible beneficiaries aged 65 years and older at private sector medical facilities, over 91% of health care encounters among non-service member beneficiaries (TRICARE-eligible and Medicare-eligible) occurred at non-military medical facilities.

**What is the impact on readiness and force health protection?:**

Ensuring medical care for family members, especially during deployments or other periods of separation, improves service member focus and morale. The promise of lifetime medical benefits upon retirement for not only service members but their immediate family members has been a powerful recruitment and retention tool over the past several decades. Moreover, health care provision to beneficiaries who are not active duty service members provides Military Health System care providers with valuable opportunities for maintaining their requisite medical skills and knowledge, thereby improving medical force readiness. Routinely documented and reported trends in health care utilization and diagnostic patterns can help senior leaders improve resource allocation within the Military Health System to maximize efficiency, medical readiness, and readiness of the medical forces.

## 1 BACKGROUND

The Military Health System (MHS) is a global, integrated health delivery system tasked with ensuring the medical readiness of the U.S. Armed Forces while fulfilling the individual health care needs of eligible military personnel and their dependents.^1^ The MHS network comprises military hospitals and clinics that ensure the medical readiness of the force, which are complemented by programs that enable beneficiary care in the private sector through the TRICARE insurance program.

MHS beneficiaries are a diverse and heterogeneous population that includes not only active duty service members, but retirees in addition to immediate family members, from all branches of military service under the authority of the Department of Defense.^2^ Each MHS beneficiary category has its own demographic, enrollment, and health care provision patterns. Since the TRICARE brand name was first applied to the MHS enterprise in 1995, the TRICARE benefit has continued to expand and evolve for service members, retirees, and their families. In fiscal year 2022, TRICARE served approximately 9.6 million beneficiaries.^3^

An important element of beneficiary care is the transition from TRICARE to Medicare. Once an individual reaches age 65, and becomes eligible for Medicare, TRICARE eligibility ends. If individuals enroll in Medicare, they receive a Medicare gap insurance, known as TRICARE for Life (TFL).^2^ TFL is funded through mechanisms outside of the Defense Health Program. While TFL patients are eligible for direct care at military hospitals and clinics, most care is provided at private sector institutions, paid through the Medicare benefit. While Medicare-eligible individuals remain eligible for direct care at military medical facilities, such care is contingent upon resource availability.

Beneficiaries enrolled in either the TRICARE Prime or TRICARE Select options, including many family members of active duty service members and a portion of non-Medicare eligible retirees and their family members (primarily those aged 64 years and younger), may receive care at fixed military hospitals and clinics or from private sector health care facilities (purchased care) that supplement direct military medical care. Consequently, distribution of health care burden estimates should be considered in relation to beneficiary age category and source of care when interpreting health care provision data among MHS beneficiaries.

This report represents an updated summary of health care burdens among MHS non-service member beneficiaries during calendar year 2023. Health care burdens were quantified using a classification system derived from the Global Burden of Disease (GBD) Study,^4,5,6,7^ in combination with diagnostic groupings from the International Classification of Diseases, 10th Revision, Clinical Modification (ICD-10-CM) chapter-based system for categorizing hospitalizations and ambulatory visits. This report presents stratified estimates for 4 age groups of health care recipients, and Medicare-eligible beneficiaries (over age 65) are considered separately, as most of their care is provided and paid by non-MHS resources.

## 2 METHODS

The surveillance population included all non-service member MHS beneficiaries who had at least 1 hospitalization or outpatient medical encounter from January 1 through December 31, 2023, with either a military hospital, clinic, or health care provider, or through a private sector facility or provider (if reimbursed through TRICARE or through Medicare with a copayment by TFL). All inpatient and outpatient medical encounters for this analysis were summarized according to the primary (first-listed) International Classification of Diseases, 10th Revision (ICD-10) codes that indicate the natures of illnesses or injuries (i.e., ICD-10 codes A00–T88). Nearly all records of encounters with first-listed diagnoses coded with “Z” (care other than for a current illness or injury, e.g., general medical examinations, after care, vaccinations) or “V,” “W,” “X,” or “Y” (indicators of the external causes but not the natures of injuries) were excluded from the analysis; encounters with a code of Z37 (“outcome of delivery, single liveborn”) in the primary position were retained.

For summary purposes, all illness- and injury-specific diagnoses (as defined by ICD-10) were grouped into 153 burden of disease-related conditions and 25 major morbidity categories based upon a modified version of the classification system developed for the Global Burden of Disease Study. The methodology for summarizing absolute and relative morbidity has been used annually since 2014 and is described elsewhere.^8^ Results were stratified by source of health care (direct care, i.e., military hospitals and clinics vs. non-direct care, i.e., private sector medical facilities) and by age group (0-17 years, 18-44 years, 45-64 years, 65 years and older). For analysis of morbidity burdens within the youngest age group, developmental disorders were included in the general category of mental health disorders.

## 3 RESULTS

In 2023 the population of non-service member MHS care recipients included more female (57.0%) than male (43.0%) beneficiaries. Adults ages 65 years and older accounted for the highest number of individuals receiving health care (n=2.03 million; 32.9%), followed by pediatric beneficiaries 17 years of age and younger (n=1.48 million; 24.1%), adults 18-44 years old (n=1.32 million; 21.5%), and older adults 45-64 years of age (n=1.32 million; 21.5%) (**Table 1**).

A total of 6,155,668 non-service member MHS beneficiaries had 90,192,185 recorded medical encounters in 2023. Over half (51.4%) of the medical encounters in 2023 within the MHS were among the 2,025,803 beneficiaries over age 65 (**Table 1**). Among TRICARE-eligible beneficiaries (under age 65 years), the 3 most frequent morbidity-related categories that accounted for the most medical encounters were mental health disorders, signs or symptoms of ill-defined conditions, and injury or poisoning (**Figure 1a**). While mental health disorders and injury each also accounted for 1 of the 3 highest morbidity-related groupings for hospital bed days, maternal conditions accounted for the second-highest number of bed days among beneficiaries under age 65 years (**Figure 1b**).

**Pediatric beneficiaries, ages 17 and younger**

Pediatric patients accounted for 14.9% of all medical encounters, 24.1% of all individuals affected, and 8.3% of all hospital bed days (**Table 1**) among non-service member MHS beneficiaries in 2023. On average, each pediatric beneficiary had 9 medical encounters during the year. Among TRICARE-eligible beneficiaries (excluding Medicare-eligible beneficiaries 65 and older), this group accounted for 30.6% of medical encounters, 35.9% of individuals affected, and 26.4% of all bed days.

Mental health disorders represented the largest burden of disease in 2023 among pediatric beneficiary medical encounters (37.7%; n= 5,064,319) and contributed to the highest number of hospital bed stays (59.2%; n=300,462) (**Figures 2a** and **2b**). On average, pediatric beneficiaries affected by a mental health disorder experienced 15 medical encounters during the year specifically related to this morbidity category (data not shown). More than two-thirds (68.2%) of all medical encounters for mental health disorders among pediatric beneficiaries were attributed to 3 groups of disorders: autism-related disorders (32.2%), developmental disorders of speech and language (25.4%), and attention-deficit hyperactivity disorders (10.6%) (**Figure 2c**). Pediatric patients affected by an autistic disorder had, on average, 41 autism-related encounters per individual (data not shown). Despite the high numbers of encounters associated with these 3 categories of mental health disorders, approximately three-quarters (70.4%) of hospital bed days related to mental health disorders were attributable to mood disorders, and 29.2% of bed days related to mood disorders were attributable to “disruptive mood dysregulation disorder (ICD10: F3481)” (data not shown).

Perinatal conditions, or medical issues occurring within 1 year of birth, accounted for the second highest number of hospital bed days (n=42,941; 8.5%) among pediatric beneficiaries, after mental health disorders (**Figures 2a** and **2b**), in 2023. Among pediatric beneficiaries with at least 1 illness- or injury-related diagnosis, those with malignant neoplasms had the second highest number (14) of related encounters per affected individual. The highest numbers of malignant neoplasm-related encounters and hospital bed days were attributable to leukemias (data not shown).

Respiratory infections (including upper and lower respiratory infections and otitis media) accounted for more medical encounters among pediatric beneficiaries (10.5%) compared to any older age group of beneficiaries (**Figures 2b**,**3b**,**4b**,**5b**). Pediatric beneficiaries accounted for a smaller proportion (3.4%) of bed days due to respiratory disease than adults ages 65 years and older (6.3%).

**Beneficiaries ages 18 to 44**

Non-service member beneficiaries ages 18-44 years accounted for 14.4% of all medical encounters, 21.5% of all individuals affected, and 10.0% of hospital bed days in 2023 (**Table 1**). On average, each individual who was 18-44 years of age and affected with an illness or injury (any cause) had 10 medical encounters during the year.

Mental health disorders accounted for the most medical encounters (n=3,210,427; 24.8% of all encounters) among beneficiaries ages 18-44 in 2023 (**Figures 3a** and **3b**), representing over one-fifth (21.6%) of total bed days, and on average, 8 mental health disorder-related encounters per individual during the year. Anxiety disorders (35.3%), mood disorders (30.2%), and adjustment disorders (14.9%) accounted for approximately four-fifths (80.4%) of all medical mental health disorder encounters among beneficiaries ages 18-44 years (data not shown). Among adult beneficiaries in this age group, mood and substance abuse disorders accounted for over three-quarters (50.0% and 26.7%, respectively) of hospital bed days for mental health disorders.

Maternal conditions accounted for more than two-fifths (44.2%) of all bed days among adults ages 18-44 years as well as, on average, 7 medical encounters per affected individual (**Figures 3a** and **3b**). Infant deliveries accounted for 9.6% of maternal condition-related medical encounters (data not shown).

Malignant neoplasms, as a diagnostic group, resulted in 6.7 encounters on average per individual in 2023, and were relatively comparable to maternal conditions, which resulted in 6.6 encounters per individual, in addition to mental health disorders, at 8.3 encounters per individual. Of the 101,474 medical encounters for malignant neoplasms among adults ages 18-44 years, 32.9% were attributed to malignant neoplasm of the breast (data not shown).

**Beneficiaries ages 45 to 64**

Non-service member beneficiaries ages 45-64 years accounted for approximately one-fifth (19.3%) of all medical encounters, 21.5% of all individuals affected, and 13.2% of hospital bed days in 2023 (**Table 1**). Each affected individual ages 45-64 years necessitated, on average, 13 medical encounters during the year. Of all morbidity-related categories, musculoskeletal diseases accounted for the most medical encounters (n=2,516,572; 14.4%) among older adult beneficiaries ages 45-64 years (**Figures 4a** and **4b**); back problems accounted for 42% of these musculoskeletal disease-related encounters (data not shown).

Cardiovascular diseases represented the highest proportion of hospital bed days (17.0%), second to injury or poisoning (16.4%) among adults ages 45-64 years (data not shown). Digestive diseases (9.4%) and malignant neoplasms (7.9%) accounted for larger percentages of total hospital bed days among beneficiaries of this age group than within other age groups of non-service member MHS beneficiaries.

Malignant neoplasm of the breast was the leading cause of neoplasm-related medical encounters (25.4%) by adults ages 45-64 years who were non-service member beneficiaries of the MHS in 2023 (data not shown).

**Medicare-eligible beneficiaries ages 65 and older**

Non-service member beneficiaries ages 65 years and older accounted for more medical encounters (51.4%) and more than 2.2 times the number of hospital bed days in 2023 than all other age groups combined. On average, each affected individual in this age group necessitated 23 medical encounters during the year. In addition, the number of individuals affected in this age group was greater than in any other age group (**Table 1**).

Musculoskeletal diseases (n=6,869,432; 14.8%) together with cardiovascular diseases (n=6,285,255; 13.6%) represented the leading causes for medical encounters among beneficiaries ages 65 years and older, while cardiovascular diseases, as a discrete cause, accounted for the second largest number of bed days (850,261 days; 20.4%) in 2023 (**Figures 5a** and **5b**). Back problems accounted for a little more than one-third (35.4%) of all musculoskeletal disease-related medical encounters among non-service member MHS beneficiaries in 2023 (data not shown).

## 4 DISCUSSION

This report documents the overall health care burden of disease among non-service member MHS beneficiaries in 2023, received through not only direct care at military hospitals and clinics, but purchased care reimbursements at private sector medical facilities as well. A substantial majority of non-service member beneficiaries received care exclusively at private sector facilities: Just under 8% of all ambulatory encounters documented in the Defense Medical Surveillance System (DMSS) in 2023 were provided from a direct care military hospital or clinic. This finding also reflects the substantial proportion of encounters and bed days among non-service member beneficiaries that is attributable to Medicare-eligible (65 years and older) beneficiaries.

The total number of non-service member beneficiaries receiving health care through the MHS in 2023 was 6,155,668, a decrease of 388,365 from 6,544,033 in 2022. Unlike in 2022, the number of pediatric beneficiaries under age 17 years exceeded those ages 18 to 44 years (n=1.48 million, 24.1% vs. n=1.32 million, 21.5%, respectively). Pronounced differences between beneficiary age groups exist for types of morbidity-related diagnoses and disease-specific conditions. Individuals ages 65 years or older—32.9% of all non-service member beneficiaries receiving an illness-or injury-specific diagnosis in 2023—accounted for approximately half (51.4%) of all medical encounters and over two-thirds (68.5%) of all hospital bed days among all beneficiaries.

The National Ambulatory Medical Care Survey of 2019 documented a substantially lower rate of ambulatory visits (3.2 visits per person-year)^9^ among the general U.S. population than among non-service member MHS beneficiaries (14.7 visits per person-year) reported here. This higher rate of ambulatory visits among non-service member beneficiaries compared to national civilian data was observed for all age groups. Future analyses comparing major diagnostic category rates to civilian counterparts, by age and sex, may be useful for identifying longitudinal morbidity outcomes unique to military service. Since the National Ambulatory Medical Care survey includes uninsured individuals, financial barriers to care may explain a portion of the lower overall utilization rate among the general U.S. population, while the families of uniformed personnel require more medical procedures in practice, which is reflected in the composition of the most common directly-provided and purchased procedures.^10,11^

As in previous years, mental health disorders were the leading cause in 2023 for medical encounters within the pediatric (ages 0-17 years) and young adult (ages 18-44 years) beneficiary age groups, although the proportion of medical encounters attributed to mental health disorders was markedly lower among young adult (24.8%) than pediatric (37.7%) beneficiaries. Developmental disorders were a significant factor for pediatric beneficiary health care, with 68% of medical encounters for mental health disorders attributable to autism-related disorders, specific developmental disorders of speech and language, or attention-deficit hyperactivity disorders.

While ambulatory encounters among non-service member beneficiaries in 2023 remained relatively stable (with only a 2% increase) compared to 2022, the crude annual difference in hospital bed days decreased by 14%. The decreasing trend for bed days was influenced by beneficiaries ages 65 years and older, in particular their hospital bed days for cardiovascular disease, which reduced to 850,261 in 2023 from 1,201,613 in 2022, a 41% decrease. Meanwhile, the number of individuals ages 65 years or older affected by cardiovascular disease remained stable, decreasing slightly from 1,213,404 individuals in 2022 to 1,209,734 individuals in 2023. Since this report does not include person-time nor approximate rates, annual comparisons are not proportionate to changes in the number of beneficiaries procuring care. Further investigation of this finding may be of interest to MHS researchers, however, as it is noteworthy that the number of hospital days for beneficiaries ages 65 years and older decreased dramatically, along with a decrease in hospitalization days due to cardiovascular disease in 2023. Additional research is needed to determine the actual cause of the decline in cardiovascular disease and assimilate those findings to further improve case and condition management.

Cardiovascular diseases, which accounted for the largest portion of hospitalization days, along with malignant neoplasms of the breast, which represent moderate rates (32.9% vs. 25.4%) among adults ages 18-44 years and 45-64 years, respectively, continue to present as a serious burden to non-service member recipients each year. Both diseases involve many risk factors that are preventable, such as obesity and smoking. Further attention on effective prevention methods is necessary to effectively manage these diseases.^12^

As the MHS completes its transition to the new MHS GENESIS electronic health record, AFHSD is also in the process of completely transferring or mapping electronic health record data to the DMSS. 2023 was the second year DMSS data were housed and analyzed from the new MHS Information Platform (MIP). During this transition to the MIP, the number of records transmitted from MHS GENESIS and the Tricare Encounter Detail to DMSS are being continually reviewed for completeness of data capture.

While this report aims to describe morbidity-related diagnoses for all MHS beneficiaries, the data are limited to beneficiaries who received care at military hospitals and clinics or at private sector medical facilities and reimbursed through TRICARE (as primary or secondary insurance) or through Medicare, if TFL was also billed. Certain forms of care provision, such as that paid with other health insurance and not billed to TRICARE, or paid directly by the patient (or family member), is not captured in this report.

The Military Health System Strategy for Fiscal Years 2024-2029 calls for additional capacity, to facilitate the return of patients including non-service member beneficiaries to military hospitals and clinics, improve their access to care, and increase opportunities for sustaining military clinical readiness for medical forces while delivering quality care to beneficiaries.^1,13^ The need to “attract and reattract” beneficiaries to the direct care setting may be reflected in the data throughout this report, which indicate a substantial proportion of medical encounters and hospitalizations for non-service member MHS beneficiaries exclusively from private sector care. Continued evaluation of health care utilization, provision, and diagnostic patterns may aid senior leaders’ allocation of resources for realization of the current MHS strategy and goals.

## Figures and Tables

**Table:** Medical Encounters^a^, Individuals Affected^b^, and Hospital Bed Days by Source and Age Group, MHS Non-Service Member Beneficiaries, 2023

	Medical Encounters		Individuals Affected		Medical Encounters per Individual Affected	Hospital Bed Days	
	No.	% Total	No.	% Total		No.	% Total
All non-service member beneficiaries	90,192,185	---	6,155,668	---	14.7	6,083,009	---
**Source**							
Direct care only	6,922,949	7.7	463,593	7.5	14.9	270,571	4.4
Outsourced care only^c^	83,269,236	92.3	4,715,955	76.6	17.7	5,812,438	95.6
Direct and outsourced care^d^	N/A	N/A	976,120	15.9	N/A	N/A	N/A
**Age group, y**							
0–17	13,426,358	14.9	1,482,338	24.1	9.1	507,252	8.3
18–44	12,970,904	14.4	1,323,647	21.5	9.8	607,208	10.0
45–64	17,418,985	19.3	1,321,940	21.5	13.2	804,712	13.2
65+	46,344,941	51.4	2,025,083	32.9	22.9	4,163,837	68.5

Abbreviations: MHS, Military Health System; No., number; N/A, not applicable; y, years.

^*^ A total of 2,660 individuals of unknown age with their medical encounters in this group, 30,997, were not included in age-specific categorical analyses but were included in the overall total.

^a^ Medical encounters include total hospitalizations and ambulatory visits for the condition (with no more than 1 encounter per individual per day per condition).

^b^ Individuals with at least 1 hospitalization or ambulatory visit for the condition.

^c^ Represents encounters or hospital bed days under purchased care or care received under Medicare benefit.

^d^ Represents a combination of care received directly at military hospitals and clinics and non-military medical facilities.

**Figure 1a:**
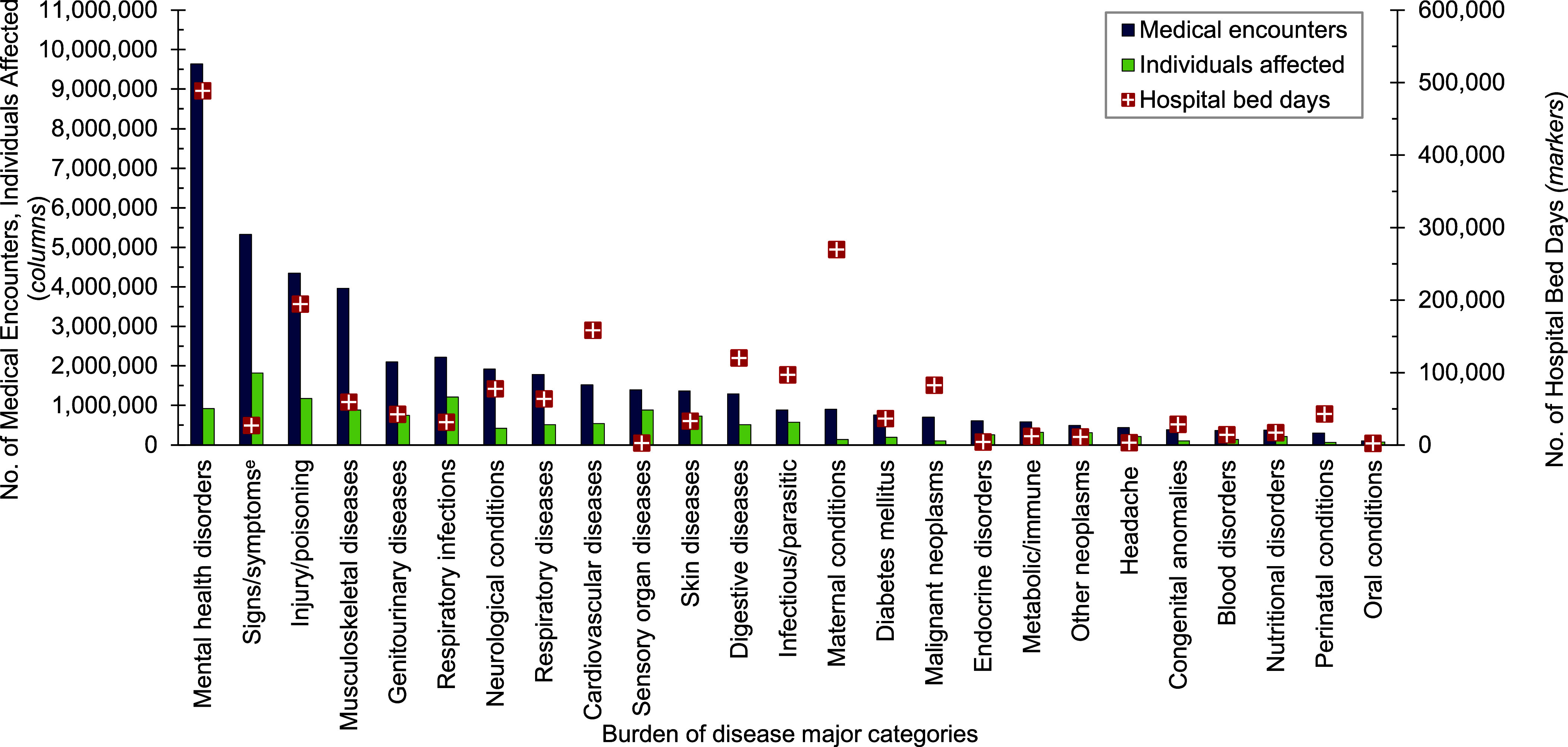
Numbers of Medical Encounters^a^, Individuals Affected^b^, and Hospital Bed Days by Burden of Disease Major Category^c^, MHS Non-Service Member Beneficiaries Under Age 65 Years^d^, 2023

**Figure 1b:**
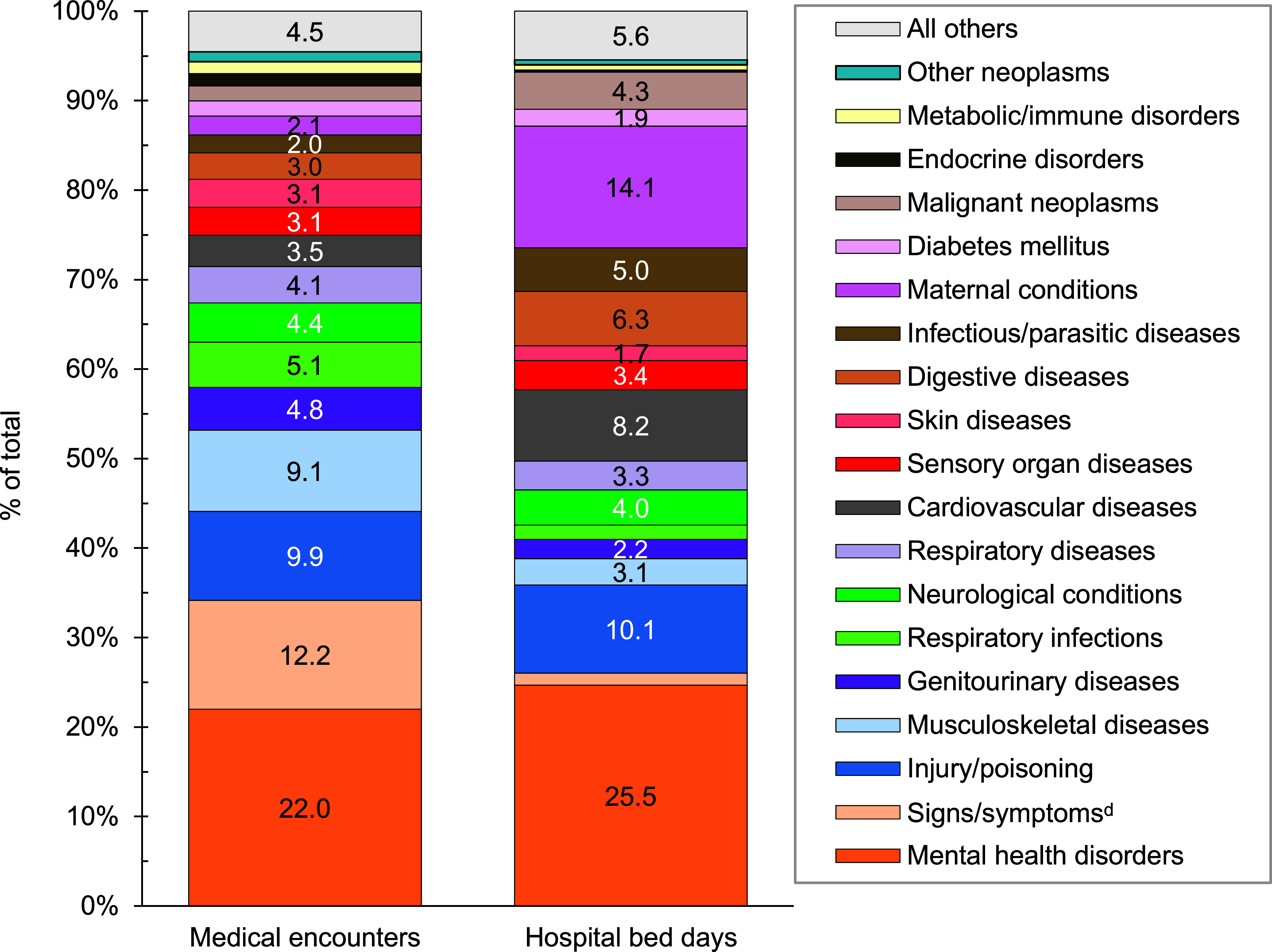
Percentages of Medical Encounters^a^ and Hospital Bed Days by Burden of Disease Major Category^b^, MHS Non-Service Member Beneficiaries Under Age 65 Years^c^, 2023

**Figure 2a:**
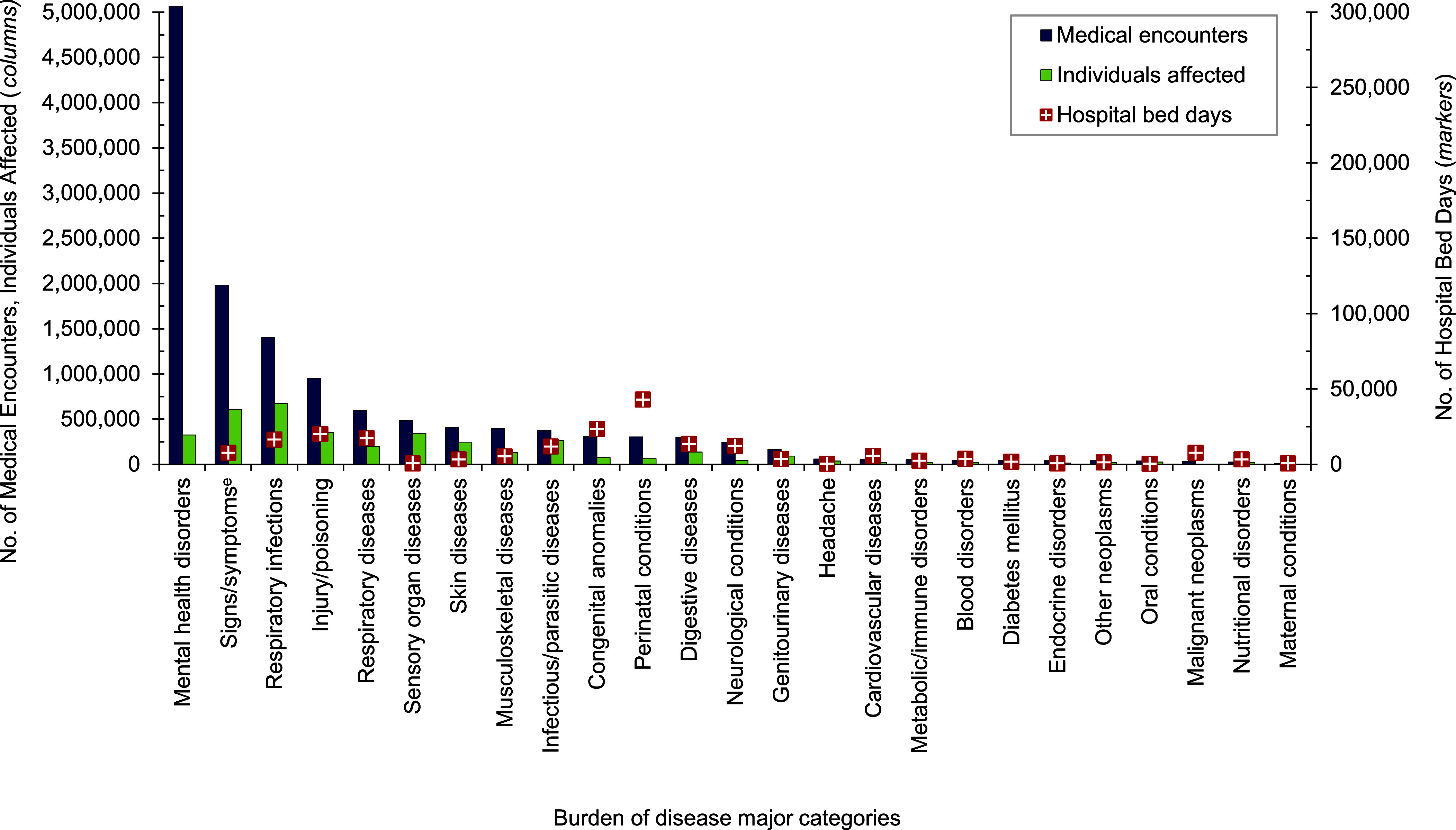
Medical Encounters^a^, Individuals Affected^b^, and Hospital Bed Days by Burden of Disease Major Category^c^, MHS Pediatric Beneficiaries^d^, Ages 17 Years and Younger, 2023

**Figure 2b:**
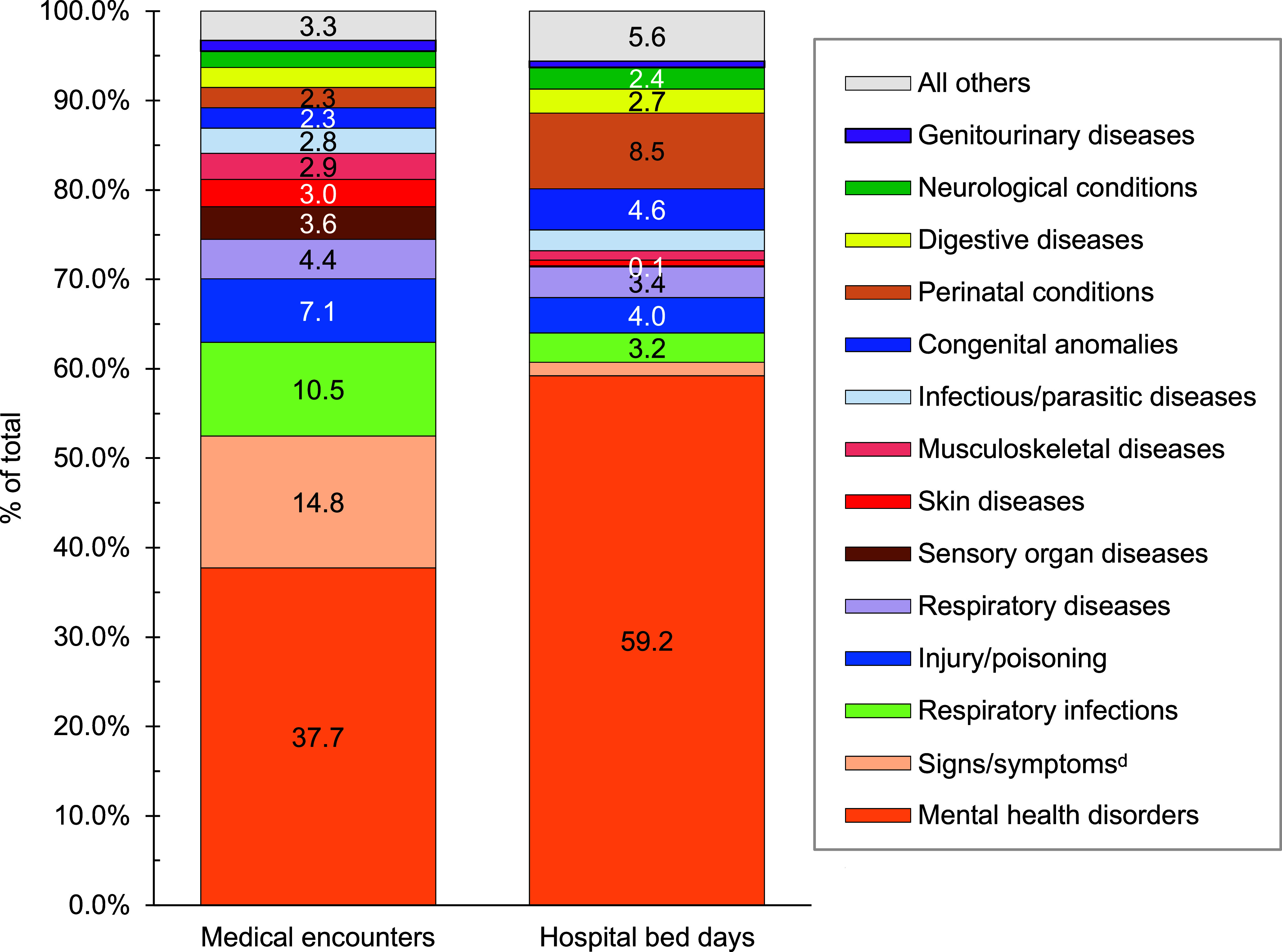
Percentages of Medical Encounters^a^ and Hospital Bed Days by Burden of Disease Category^b^, MHS Pediatric Beneficiaries^c^, Ages 17 Years and Younger, 2023

**Figure 2c:**
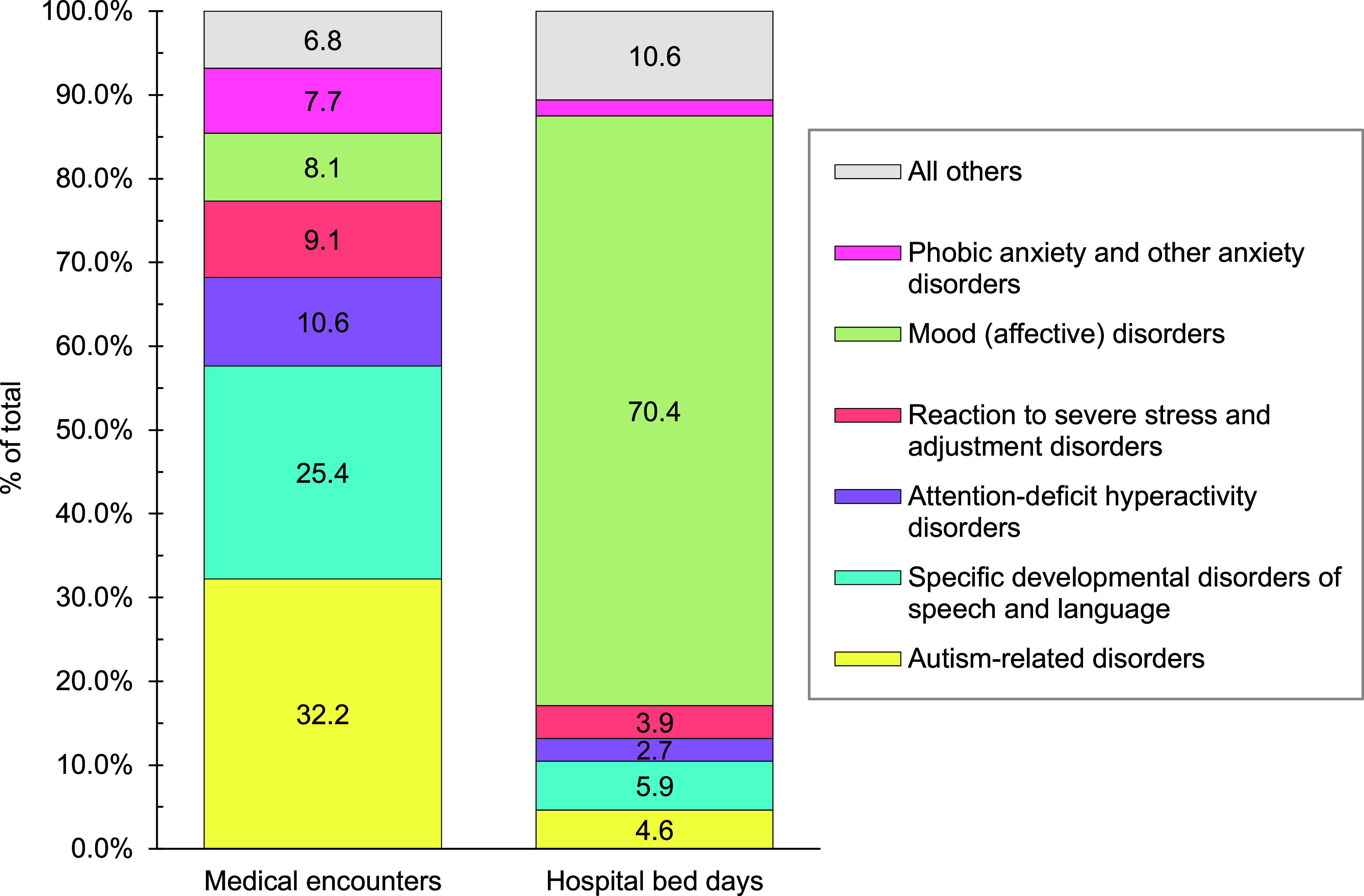
Percentages of Medical Encounters^a^ and Hospital Bed Days for Mental Health Disorders by Conditions with Greatest Morbidity Burdens, MHS Pediatric Beneficiaries, Ages 17 Years and Younger, 2023

**Figure 3a:**
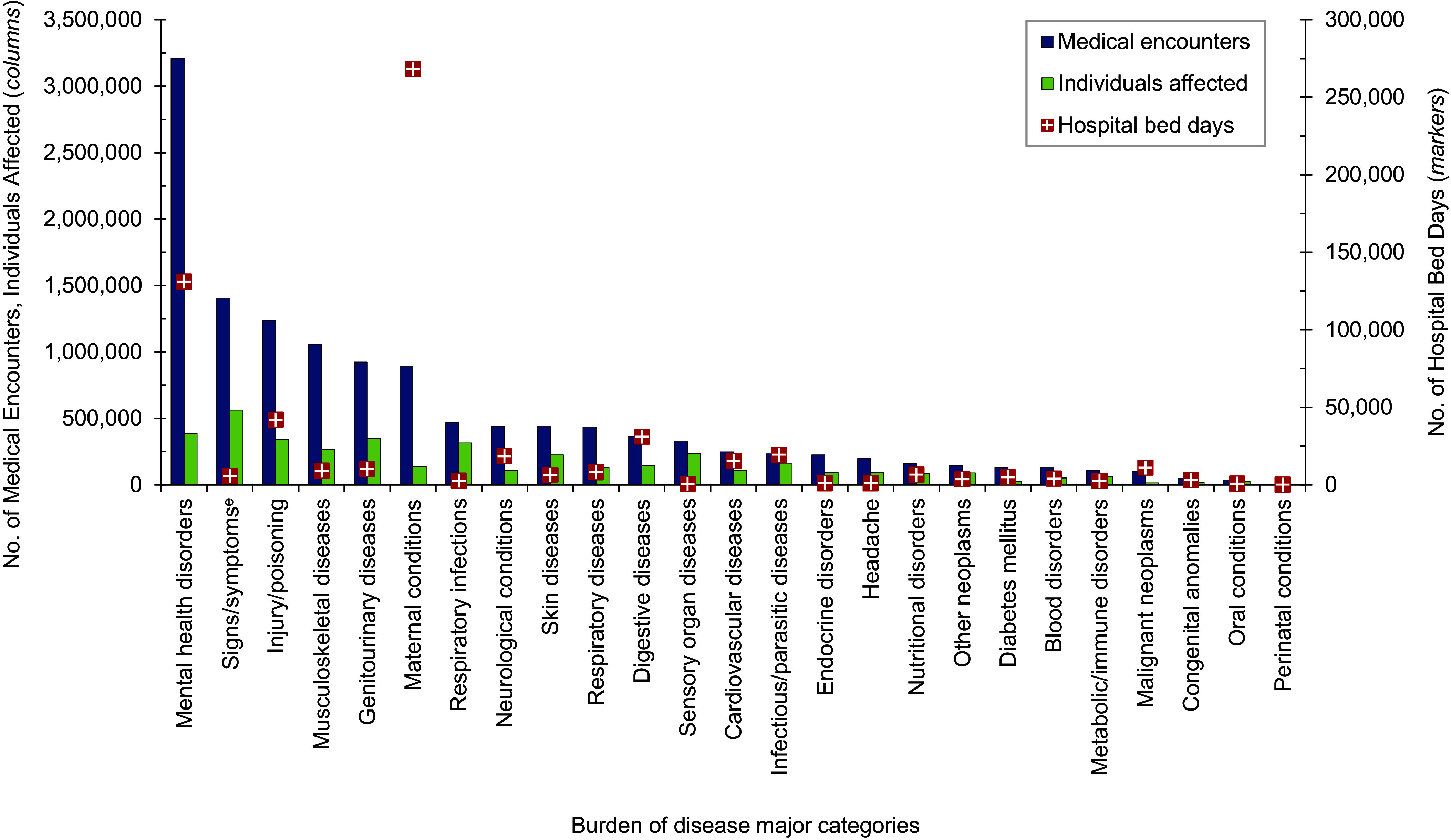
Medical Encounters^a^, Individuals Affected^b^, and Hospital Bed Days by Burden of Disease Major Category^c^, MHS Non-Service Member Beneficiaries^d^, Ages 18–44 Years, 2023

**Figure 3b:**
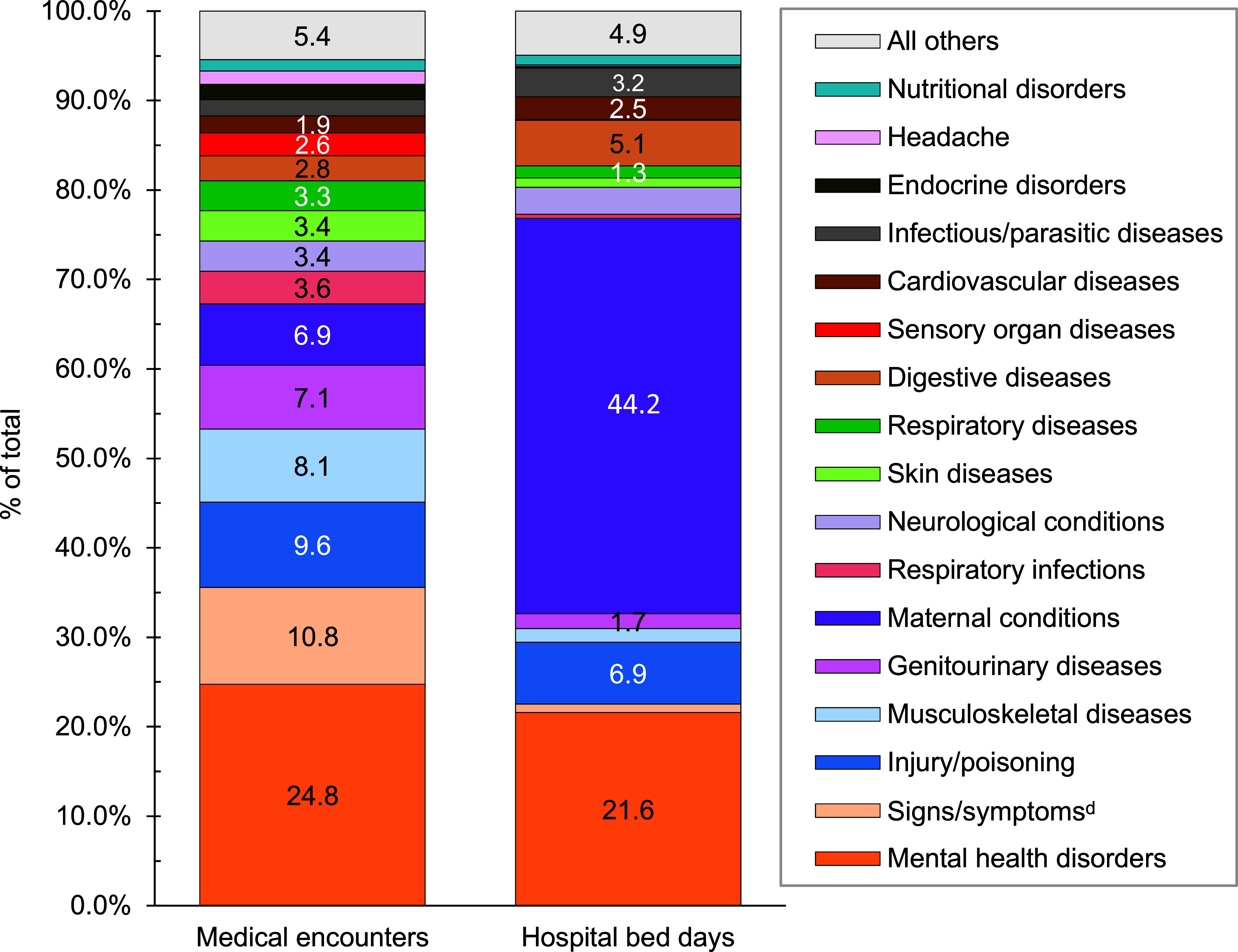
Percentages of Medical Encounters^a^ and Hospital Bed Days, by Burden of Disease Major Category^b^, MHS Non-Service Member Beneficiaries^c^, Ages 18–44 Years, 2023

**Figure 4a:**
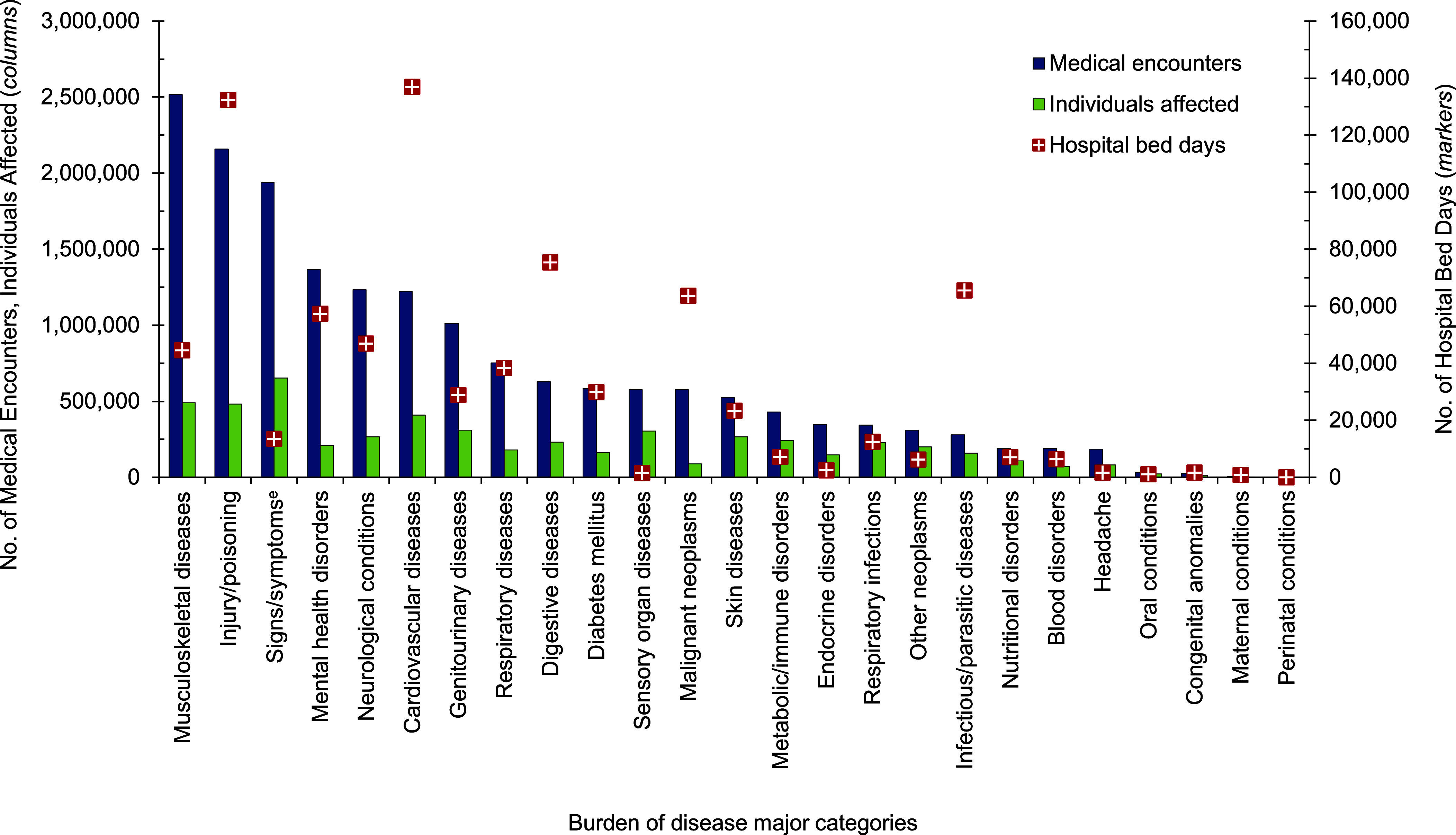
Medical Encounters^a^, Individuals Affected^b^, and Hospital Bed Days by Burden of Disease Major Category^c^, MHS Non-Service Member Beneficiaries^d^, Ages 45–64 Years, 2023

**Figure 4b:**
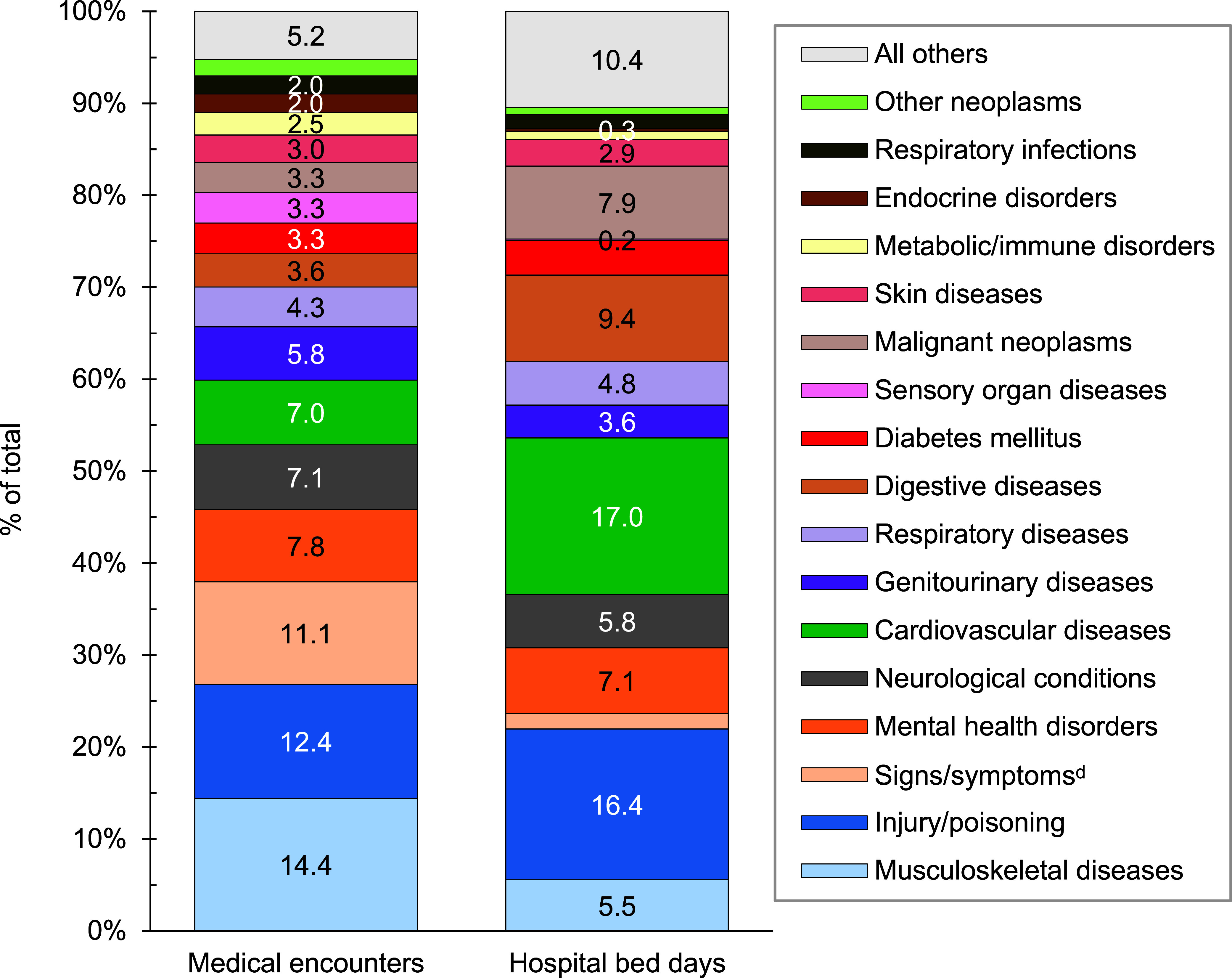
Percentages of Medical Encounters^a^ and Hospital Bed Days by Burden of Disease Major Category^b^, MHS Non-Service Member Beneficiaries^c^, Ages 45–64 Years, 2023

**Figure 5a:**
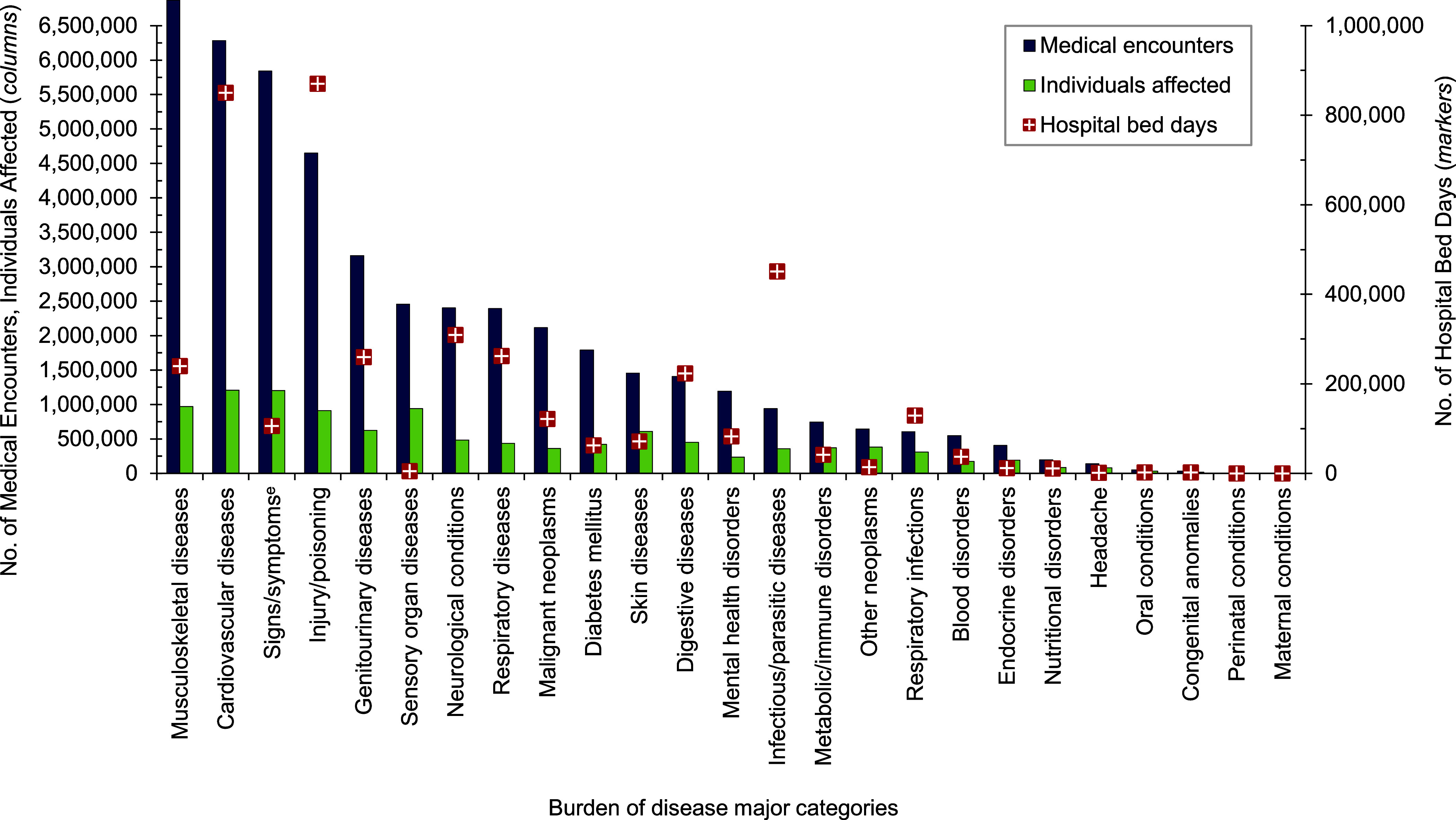
Medical Encounters^a^, Individuals Affected^b^, and Hospital Bed Days by Burden of Disease Major Category^c^, MHS Non-Service Member Beneficiaries^d^, Ages 65 Years and Older, 2023

**Figure 5b:**
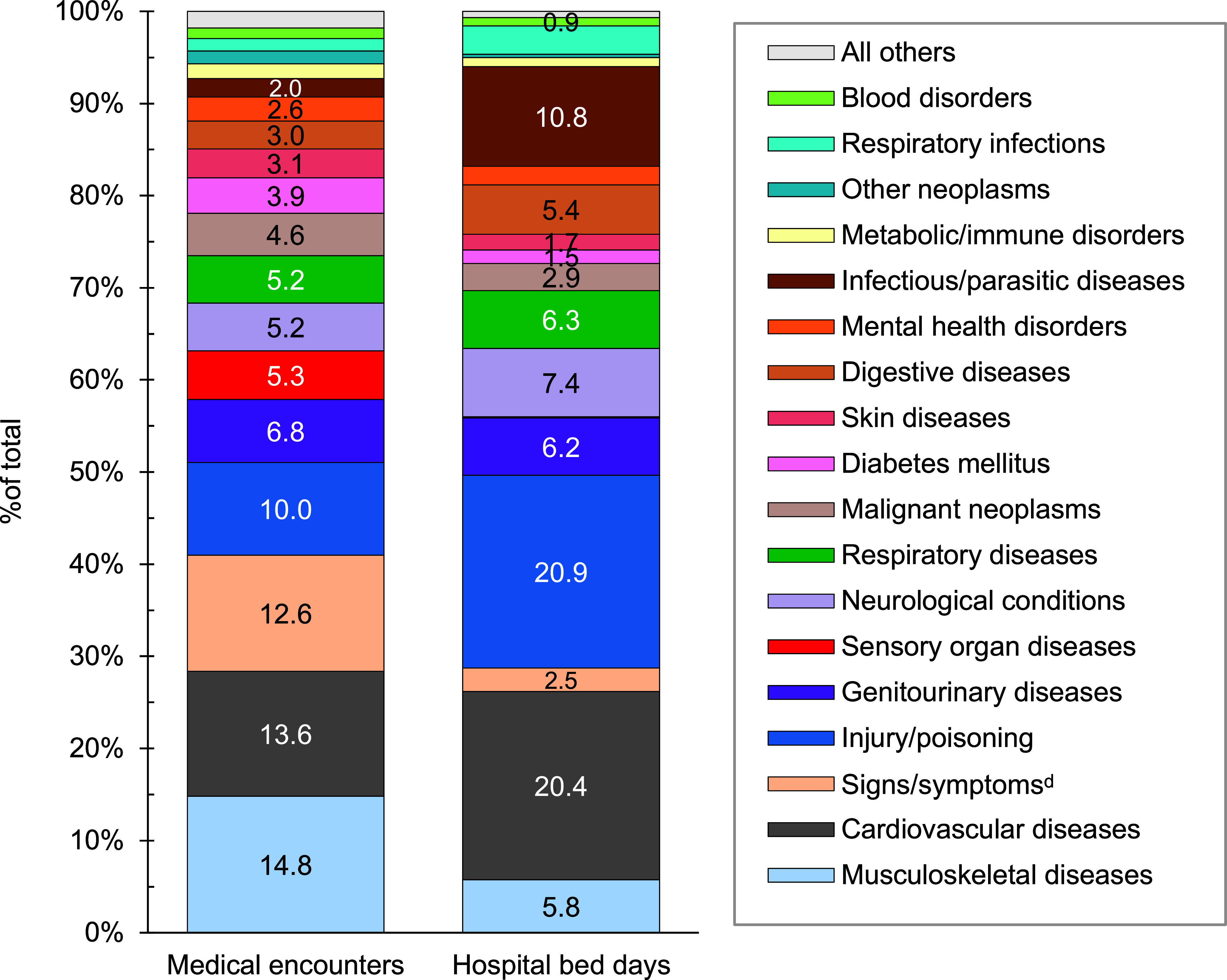
Percentages of Medical Encounters^a^ and Hospital Bed Days by Burden of Disease Major Category^b^, MHS Non-Service Member Beneficiaries^c^, Ages 65 Years and Older, 2023
